# Alcohol use: prevalence and associated factors in a sample of Tunisian students

**DOI:** 10.1192/j.eurpsy.2023.1406

**Published:** 2023-07-19

**Authors:** R. Ben Soussia, N. Faouel, W. Bouali, E. Gharbi, M. Ben Fredj, L. Zarrouk

**Affiliations:** 1 Tahar Sfar Mahdia Hospital, Mahdia, Tunisia; 2 Psychiatry Hospital, Tahar Sfar Mahdia Hospital, Mahdia; 3 Hôpital fattouma Bourguiba Monastir, Monastir, Tunisia

## Abstract

**Introduction:**

The use of alcohol, in particular its harmful use and alcohol dependence, represent a public health problem of interest to all age groups. Indeed, alcohol is responsible for a heavy burden of disease and a considerable socio-economic burden.

**Objectives:**

To determine the prevalence of alcohol use and the main factors associated with it in a sample of Tunisian students.

**Methods:**

This is an analytical cross-sectional study carried out during the 2020/2021 academic year with a sample of Tunisian students. We used an anonymous self-administered questionnaire distributed online via social networks. Our questionnaire included a section focusing on socio-demographic characteristics and the AUDIT test (Alcohol Use Disorders Identification Test) to detect alcohol addiction.

**Results:**

Our study enrolled 772 students .The average age of the study population was 23.29 ±3.25.Two hundred ninety-nine students (38.7%) consumed alcohol. The mean age at first use was 18.44 (3.09). Fifty-two alcohol users (17.4%) presented a risk of alcohol dependence. The first consumption contexts were mostly “between friends” (57.4%) and “party time “(23.5%).The main effects sought were the effect of disinhibition (64.5%) and social integration (41.1%) were predominant responses. The determining factors of alcohol consumption were age (p<10^-3^), male gender (p<10^-3^), and the presence of grade repetition (p<10^-3^), wealthy socioeconomic level (p<10^-3^), and participation in community life (p<10^-3^).

**Conclusions:**

Alcohol use and especially alcohol dependence may have harmful effects on student’s life. It is relevant to screen this addiction to better its prevention.

**Disclosure of Interest:**

None Declared

